# The contribution of phenotypic plasticity to the evolution of insecticide tolerance in amphibian populations

**DOI:** 10.1111/eva.12267

**Published:** 2015-05-27

**Authors:** Jessica Hua, Devin K Jones, Brian M Mattes, Rickey D Cothran, Rick A Relyea, Jason T Hoverman

**Affiliations:** 1 Department of Forestry and Natural Resources, Purdue UniversityWest Lafayette, IN, USA; 2 Department of Biological Sciences, Rensselaer Polytechnic InstituteTroy, NY, USA; 3 Department of Biological Sciences, Southwestern Oklahoma State UniversityWeatherford, OK, USA

**Keywords:** acetylcholine esterase inhibitor, amphibian declines, genetic accommodation, *Lithobates sylvaticus*, phenotypic plasticity, toxicology

## Abstract

Understanding population responses to rapid environmental changes caused by anthropogenic activities, such as pesticides, is a research frontier. Genetic assimilation (GA), a process initiated by phenotypic plasticity, is one mechanism potentially influencing evolutionary responses to novel environments. While theoretical and laboratory research suggests that GA has the potential to influence evolutionary trajectories, few studies have assessed its role in the evolution of wild populations experiencing novel environments. Using the insecticide, carbaryl, and 15 wood frog populations distributed across an agricultural gradient, we tested whether GA contributed to the evolution of pesticide tolerance. First, we investigated the evidence for evolved tolerance to carbaryl and discovered that population-level patterns of tolerance were consistent with evolutionary responses to pesticides; wood frog populations living closer to agriculture were more tolerant than populations living far from agriculture. Next, we tested the potential role of GA in the evolution of pesticide tolerance by assessing whether patterns of tolerance were consistent with theoretical predictions. We found that populations close to agriculture displayed constitutive tolerance to carbaryl whereas populations far from agriculture had low naïve tolerance but high magnitudes of induced tolerance. These results suggest GA could play a role in evolutionary responses to novel environments in nature.

## Introduction

Human activities have dramatically altered the environment through climate change, habitat fragmentation, introduced species, and pollution (Goudie 2005). A significant concern is how populations will respond to such rapid and novel environmental changes (Sutherland et al. 2013). The traditional paradigm predicts that novel environments can enact selection upon existing constitutive traits (i.e. a trait that is constantly expressed regardless of environment; Pigliucci et al. 2006) driving populations toward an optimum (Hoffmann and Sgrò 2011; Lawrence et al. 2012). However, this process depends on existing levels of genetic variation and mutation rates, which often can limit evolution (Le Rouzic and Carlborg 2008). Because of these limitations, there has been a surge of interest in understanding the role of phenotypic plasticity in evolutionary innovation (Pigliucci et al. 2006; Crispo 2007; Moczek et al. 2011; Wund 2012). Phenotypic plasticity, defined as the capacity of a single genotype to produce different phenotypes in different environments, represents a rapid alternative solution in response to novel environments (West-Eberhard 2003; Schlichting 2008). Novel environments can induce organisms to exhibit cryptic genetic variation that may code for adaptive traits within a single generation and allow a population to persist (Lande 2009; Bonduriansky et al. 2012). Thus, phenotypic plasticity has the potential to influence evolutionary outcomes and shape adaptations in populations that experience novel environments. Despite the unprecedented rate of environmental change, our understanding of plasticity's role in evolutionary responses to novel environmental changes remains limited in wild populations (Moczek et al. 2011).

There has been considerable controversy over whether phenotypic plasticity can facilitate adaptation to novel environments, especially in wild populations (De Jong 2005; Pigliucci et al. 2006; Crispo 2007). At the core of this controversy are two fundamental questions: 1) Does exposure to a novel environment reveal cryptic genetic variation in a population through phenotypic plasticity? and 2) Does phenotypic plasticity impede or promote evolutionary change (Braendle and Flatt 2006; Wund 2012)? Controlled laboratory experiments have routinely shown that exposure to novel environments can reveal cryptic genetic variation in populations that sets the stage for evolutionary processes to operate (Schlichting 2008). Moreover, theoretical research and laboratory selection experiments have demonstrated that phenotypic plasticity can promote evolutionary change via genetic accommodation (evolution of environmentally induced phenotypes) including the evolution of constitutive traits from initially plastic traits (i.e. genetic assimilation; Schmalhauzen 1949; Waddington 1956; West-Eberhard 2003; Crispo 2007; Moczek et al. 2011). Through the process of genetic assimilation, selection acts upon the reaction norm leading to a loss of plasticity. Thus, over time, a trait that was previously induced via an environmental cue no longer requires the cue to be expressed (i.e. constitutive expression; Pigliucci et al. 2006). While this research has demonstrated the potential role of phenotypic plasticity in evolutionary responses to novel environments, there have been relatively few attempts to determine whether it actually occurs in nature (Braendle and Flatt 2006; Moczek et al. 2011).

There are challenges to testing whether and how phenotypic plasticity contributes to evolutionary responses to novel environments in wild populations (Scoville and Pfrender 2010; Moczek et al. 2011). In particular, research can be hampered by an incomplete knowledge of the ancestral environment and the initial phenotypic responses of ancestral populations. For instance, both selection on existing constitutive trait and the loss of plasticity through genetic assimilation can result in the same evolutionary end point of constitutive trait expression. Although it is usually impossible to assess ancestral conditions (except in resurrection studies; Franks 2011), a viable solution is to use populations existing along spatial environmental gradients to infer evolutionary processes and ancestral conditions (Scoville and Pfrender 2010). By substituting space for time, one can investigate evidence for genetic assimilation by examining ancestral populations that have not consistently experienced a novel environment to determine whether they express phenotypic plasticity when exposed to a novel environment and whether derived populations that have been consistently exposed to a novel environment express constitutive traits that represent adaptations to the novel environment. While the space for time approach provides a useful proxy for assessing evolutionary responses within a single generation, multigenerational studies that track populations across time are essential next steps to corroborating discoveries that use the space for time approach (Moczek et al. 2011; Wund 2012).

Pesticides are useful tools for addressing the mechanisms underlying evolutionary processes because the agent of selection is known and populations can be easily manipulated (Mallet 1989). Moreover, pesticide use varies across the landscape from high usage in areas close to agriculture to low usage in areas far from agriculture (Declerck et al. 2006). Because spatiotemporal variation in pesticide exposure can lead to rapid environmental changes (Odenkirchen and Wente 2007), the ability to rapidly induce tolerance could play a significant role not only in population persistence within a single generation but also in the evolution of constitutive tolerance across multiple generations through genetic assimilation.

**Figure 1:**
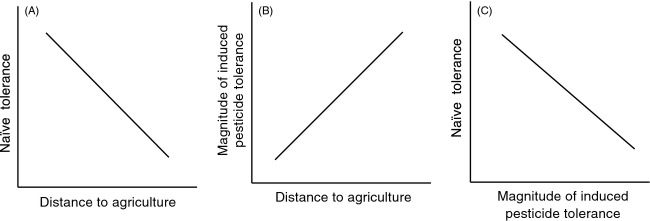
A conceptual framework for the predictions of evolved pesticide tolerance through the process of genetic assimilation. (A) If pesticides select for increased tolerance in populations over time, a negative relationship is predicted between distance to agriculture and naïve tolerance to pesticides. (B) If genetic assimilation is a mechanism for achieving the evolution of constitutive tolerance, a positive relationship is predicted between distance to agriculture and the magnitude of induced pesticide tolerance among populations. (C) If populations consistently exposed to pesticides incur costs associated with the expression of plasticity, then a negative relationship is predicted between naïve tolerance to insecticides and the magnitude of induced pesticide tolerance.

By choosing populations distributed along an agricultural gradient, it is possible to substitute space for time with populations far from agriculture representing more ancestral populations (i.e. with respect to pesticide use) and populations closer to agriculture representing derived populations (Cothran et al. 2013). We can examine individuals that have never been exposed during their lifetime (hereafter termed ‘naïve’ individuals) and individuals that have been exposed earlier in their life (hereafter termed ‘exposed’ individuals) to test the potential role of plasticity in evolutionary responses to pesticides. For instance, if pesticides select for constitutive tolerance in populations, we would predict that naïve individuals from derived populations (i.e. populations close to agriculture) should express higher naïve tolerance while individuals from ancestral populations (i.e. populations far from agriculture) should express lower naïve tolerance (Cothran et al. 2013). Thus, we would predict a negative relationship between distance to agriculture and the naïve tolerance of populations (Fig.1A). Additionally, if genetic assimilation is the mechanism underlying the evolution of constitutive tolerance, ancestral populations should express a greater magnitude of induced tolerance in response to a novel pesticide environment whereas derived populations should express a lower magnitude of induced tolerance, perhaps to the point of expressing only high levels of constitutive pesticide tolerance (Waddington 1956; Pigliucci et al. 2006; Crispo 2007; Wund 2012; Hua et al. 2013b, 2014). Thus, we would predict a positive relationship between distance to agriculture and the magnitude of induced pesticide tolerance in a population (Fig.1B). Finally, the role of genetic assimilation in evolutionary innovation (i.e. the acquisition of novel morphologies and/or behaviors that open new niches, providing new ways to successfully exploit the environment; Allen and Holling 2010) also can be evaluated by testing whether populations expressing the highest naïve tolerance express the lowest magnitudes of inducible tolerance, perhaps due to the costs associated with the expression of plasticity in constant environments (Crispo 2007). Thus, we would predict a negative relationship between the naïve tolerance of populations and the magnitude of induced pesticide tolerance (Fig.1C). Here, we test these predictions using a commonly used insecticide, carbaryl, and larval wood frogs from 15 populations distributed along an agricultural gradient.

### Study system

Ponds provide an excellent study system to explore the role of phenotypic plasticity in evolutionary innovation. Ponds are abundant, have well-defined populations, and are broadly distributed across agricultural gradients that pose variable amounts of insecticide exposure risk due to direct application and runoff (De Meester et al. 2005; Declerck et al. 2006; Gilliom 2007). Using ponds distributed along an agricultural gradient, we can assess whether patterns of pesticide tolerance are consistent with predictions of genetic assimilation. Furthermore, ponds are important habitats for many species, including amphibians. We have recently shown that nine amphibian populations vary in their constitutive tolerance to pesticides with populations living close to agriculture having higher constitutive tolerance compared to populations far from agriculture (Cothran et al. 2013). Additionally, amphibians have the ability to respond plastically to insecticides by inducing increased tolerance to lethal concentrations of insecticides following early exposure to sublethal concentrations of insecticides (Hua et al. 2013b, 2014). Using four wood frog (*Lithobates sylvaticus*) populations, Hua et al. (2013b) demonstrated that patterns of insecticide tolerance were consistent with genetic assimilation theory with the two ancestral (far from agriculture) wood frog populations exhibiting plasticity to a common insecticide by inducing increased tolerance but the two derived populations (close to agriculture) exhibiting constitutive tolerance. While our past work suggests that phenotypic plasticity and genetic assimilation played a role in evolutionary responses to pesticides, the essential next step is to expand beyond four populations to specifically test the theoretical predictions needed to provide support for the possible role of genetic assimilation in wild populations (Fig.1; Waddington 1956; Pigliucci et al. 2006; Crispo 2007; Wund 2012).

## Methods

### Insecticide background

Our focal pesticide was the insecticide carbaryl (commercial formulation Sevin©, 22.5% active ingredient CAS 63-25-2), which is a common insecticide used for pest control and disease prevention in agricultural and residential settings (Grube et al. 2011). The half-life of carbaryl at a pH of 7 is 10 days, and environmental concentrations in aquatic systems range from 0.73 to 1.5 mg/L (USEPA 2008). Carbaryl operates by reversibly binding to acetylcholine esterase (AChE) ultimately leading to the accumulation of acetylcholine and mortality (Lajmanovich et al. 2010). Reported carbaryl LC50 values for amphibians range from 1.2 to 22 mg/L (Boone and Bridges 1999; Relyea 2005).

### Animal collection and husbandry

We tested for induced tolerance to carbaryl in 15 wood frog populations collected across western Pennsylvania, USA (Table S1). We chose wood frog populations that were separated by at least 4 km. The genetic neighborhood of wood frogs is generally within ∽1 km of the breeding pond which means that we were most likely using 15 distinct populations (Berven and Grudzien 1990; Semlitsch 1998). Animals from all 15 wood frog populations were collected as early-stage embryos (Gosner 1960) within a 7-days period (Table S1). To control for the effects of developmental stage and size on sensitivity to insecticides, we manipulated temperature to standardize hatching time (Cothran et al. 2013; Hua et al. 2013a). Initially, all clutches were raised outdoors in 100-L pools filled with 90 L of aged well water (air temperature ranged from 1 to 20.6°C). On 13 April 2014, clutches collected before 7 April 2014 were chilled (1.6°C) by placing them in a walk-in cooler to slow development while clutches collected after 7 April 2014 remained outdoors in 100-L pools where they experienced warmer conditions (air temperature ranged from 10.5 to 26.1°C). After 34 h, embryonic development of clutches collected after 7 April 2014 converged with those collected before 7 April 2014 and we moved all egg masses collected prior to 7 April 2014 back into outdoor 100-L pools filled with 90 L of aged well water. On 21 April 2014, wood frogs from all 15 populations hatched within a 20-h period (Gosner stage 20).

### Distance to agriculture

To determine each population's proximity to agriculture, we measured the linear distance from each pond (at the location, egg masses were collected) to the nearest agricultural field using Google Earth (2013, v. 7.1.2). We defined an agricultural field as any plot of land from 1993 to 2013 that was used for growing crops, raising livestock and small farm animals for domestic and commercial uses, or feedlots intended for game animals. We did not differentiate among the various types of agriculture as farmers in the area rotate crops planted from year to year. For each population, we confirmed agricultural status of all fields by visiting the field and talking to landowners or local USDA agents. However, we were not able to assess the amount or frequency of pesticide application or historical land use prior to 1993. Additionally, we note that other factors, such as topography, canopy cover, water depth, and surface area, may influence the amount of pesticides that runoff into each pond (Schriever and Liess 2007). However, these additional factors would be expected to add variation to our data and potentially obscure spatial patterns of tolerance across the 15 populations.

### Experimental design overview

We tested for induced tolerance using a two-phase experiment similar to that of Hua et al. (2013b). In Phase 1, we exposed wood frog hatchlings (Gosner stage 20) from all 15 populations to either a pesticide-free control or a sublethal carbaryl treatment to induce tolerance. In Phase 2 of the experiment, we tested whether exposure to a sublethal concentration of carbaryl during the hatchling stage induced an increase in tolerance to carbaryl during the tadpole stage (Gosner stage 25). We assessed tolerance using a time to death (TTD) assay, a common measure of relative tolerance among different experimental groups (Bridges and Semlitsch 2000; Cothran et al. 2013).

### Phase 1 – Inducing higher tolerance

For all 15 populations, we haphazardly chose 300 hatchlings from each population once animals reached Gosner stage 20 on 21 April 2014. Using 14-L plastic containers as our experimental unit, we assigned 150 hatchlings from each population to 7 L of a pesticide-free control (UV-irradiated, carbon-filtered well water) or 7 L of a sublethal carbaryl solution (nominal concentration: 0.5 mg/L of carbaryl). These two groups represented our naïve and exposed tadpoles, respectively. We chose 0.5 mg/L as the sublethal concentration because past studies have demonstrated that this concentration induces tolerance without causing mortality (Hua et al. 2013b). Hatchlings were held in the laboratory at a constant temperature of 21°C on a 16:8 light dark cycle, and the insecticide solutions were not renewed. After 72 h of exposure, we transferred all individuals (Gosner 24) to 14-L containers filled with 7 L of pesticide-free well water. The hatchlings were held in clean water and were not fed because they were still living on their yolk reserves for 24 h until all individuals reached Gosner stage 25. We euthanized (MS-222 overdose) and preserved 10 randomly selected tadpoles from each treatment to assess the effect of sublethal exposure to carbaryl on tadpole mass at the end of Phase I (Appendix S1).

### Phase 2 – TTD assay: Lethal exposure to assess induced tolerance

Once tadpoles from all populations reached Gosner stage 25 on 25 April 2014, we began Phase 2 of the experiment by crossing the two Phase 1 treatments with a pesticide-free control and a lethal carbaryl treatment in the TTD assay. Our objective for the TTD assays was to cause moderate mortality over time (Newman 2010). Based on a pilot toxicity study, we chose to use 20 mg/L of carbaryl. TTD assays commonly use relatively high concentrations as a tool to assess the relative sensitivities of different groups with the expectation that these differences in mortality also provide information regarding relative differences in sublethal effects between groups (Newman 2010). Using a factorial, completely randomized design, this produced 60 treatments (15 populations × two Phase 1 treatments × two Phase 2 treatments) that were each replicated five times for a total of 300 experimental units.

The experimental units were 100-mL glass Petri dishes filled with either 70 mL of water (control) or 70 mL of the lethal carbaryl solution (20 mg/L). Keeping individuals from each population together, we haphazardly assigned 10 tadpoles to each experimental unit. We conducted water changes every 24 h with a renewal of the pesticide concentration. To assess tadpole tolerance using TTD, we monitored tadpole mortality every 2 h for the first 12 h, every 4 h after 12 h, and terminated the experiment at 96 h. In accordance with standard toxicity tests, tadpoles were not fed during the test (ASTM 2014). The tadpoles had food reserves in the form of yolk as evidenced by the low mortality observed in the pesticide-free controls from the TTD assay (0.2% mortality).

### Insecticide applications

To create working solutions, we mixed commercial grade carbaryl with UV-irradiated, carbon-filtered well water (pH = 7.5). For Phase 1, we added 15 μL of commercial grade carbaryl to 7 L of filtered water in plastic 14-L containers to achieve 0.5 mg/L of carbaryl. Hatchlings from all 15 populations were added within 10 min of dosing. For Phase 2, we added 1.185 mL of commercial grade carbaryl to 14 L of filtered water in a 45.5 L glass aquarium to achieve 20 mg/L. We then added 70 mL of the 20 mg/L carbaryl solution to each Petri dish. After adding the insecticide solutions, we added ten tadpoles to each Petri dish within 20 min of dosing. Finally, we used UV-irradiated, carbon-filtered water to create the control solutions and added 70 mL and ten tadpoles to each Petri dish within 30 min of their carbaryl counterparts.

### Insecticide testing

To determine the actual concentrations of insecticides used in this study, we collected a 500-mL sample of the 0.5 mg/L treatment after hatchlings were added during Phase 1 and a 500-mL sample of the 20 mg/L treatment after tadpoles were added into Petri dishes during Phase 2. Because we used filtered water from the same source for the control treatments in both Phase 1 and 2, we collected a single 500 mL sample from this source to be tested. All samples were sent to the University of Connecticut's Center for Environmental Sciences and Engineering (Storrs, CT). For nominal concentration 0, 0.5, and 20 mg/L, actual concentrations were 0, 0.7, and 21 mg/L, respectively (reporting limit = 0.5 μg/L).

### Statistical analysis

To explore the potential contribution of plasticity to the evolution of constitutive tolerance, we tested the following predictions: (i) the naïve tolerance of populations to carbaryl will be negatively related to distance to agriculture, (ii) the magnitude of induced tolerance will be positively related to distance to agriculture, and (iii) the magnitude of induced tolerance in the populations will be negatively related to the naïve tolerance of the populations.

For our measure of naïve pesticide tolerance for each of the populations, we focused on tadpoles that were not exposed to pesticides during Phase 1 but were exposed to carbaryl during the TTD assay of Phase 2. Naïve tolerance was calculated as the proportion of tadpoles that survived the lethal carbaryl exposure at 96 h for each of the 15 populations. We used a univariate analysis of variance (UNIANOVA; SPSS 21) to assess population-level differences in survival at 96 h. Because individuals within a Petri dish are not independent of each other, we included dish as a random effect. Also, as the normality assumption was not met, we ranked transformed the data and used SNK post hoc analysis which is an appropriate pairwise analysis for ranked cases (Quinn and Keough 2002).

To test for the presence of induced tolerance to carbaryl in the populations, we used Cox regression analyses (SPSS 21) to calculate a hazard regression coefficient (b) for each population that compared the survival of naïve individuals to the survival of exposed individuals. We conducted a separate Cox regression analysis for each population using individual tadpole time to death values with Petri dish included as a covariate (Hua et al. 2013b, 2014). The value of the coefficient indicates the relative probability that an exposed tadpole will experience mortality when exposed to a lethal concentration of carbaryl compared to naïve tadpoles (Walters 2009). When b < 0, exposed tadpoles are less likely to experience mortality from a lethal dose compared to naïve tadpoles (i.e. there is induced tolerance; Walters 2009). In contrast, when b > 0, exposed tadpoles are more likely to experience mortality from a lethal dose compared to naïve tadpoles (i.e. there is induced susceptibility).

Using our measures of naïve and induced tolerance to carbaryl, we conducted three linear regression analyses (SPSS 21) to assess the predictions of genetic assimilation theory. Our first analysis assessed the relationship between distance to agriculture and naïve tolerance (average survival at 96 h). Our second analyses assessed the relationship between distance to agriculture and the magnitude of induced tolerance to carbaryl (b). Our third analysis assessed the relationship between naïve tolerance (average survival at 96 h) and the magnitude of induced tolerance to carbaryl (b). In two populations (RR and STB), a pre-exposure induced lower tolerance (i.e. b > 0). To assess whether these populations influenced the interpretation of the regression analyses, we conducted two additional regression analyses that assessed the relationship between induced tolerance to carbaryl versus distance to agriculture and induced tolerance to carbaryl versus constitutive tolerance (average survival at 96 h) with the two populations excluded. For all analyses, as we had *a priori* predictions about the direction of ach relationship, we present just the one-tailed results (all other results reported in Table S2).

## Results

### Patterns of naïve carbaryl tolerance with distance to agriculture

**Figure 2:**
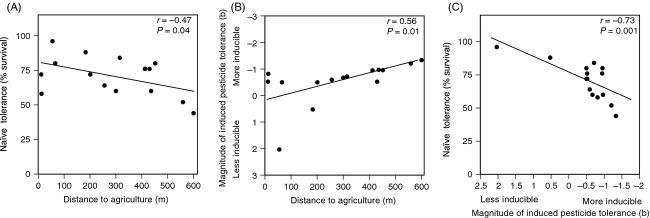
The relationships between (A) distance to agriculture and mean naïve tolerance, (B) distance to agriculture and the magnitude of induced tolerance to carbaryl, and (C) the magnitude of induced tolerance and naïve tolerance to carbaryl. All three relationships were consistent with predictions of genetic assimilation.

Wood frog populations displayed significant variation in their naïve tolerance to lethal carbaryl concentrations (*F*_14,56_ = 4.7; *P *< 0.001). Average mortality at 96 h ranged from 4% to 56% (Fig. S2). Population-level variation in naïve tolerance was negatively related to distance to agriculture (*r *= −0.47, *P* = 0.04; Fig.2A); the naïve tolerance of populations living close to agriculture was higher than populations living far from agriculture.

### Patterns of the magnitude of induced tolerance with distance to agriculture

**Figure 3:**
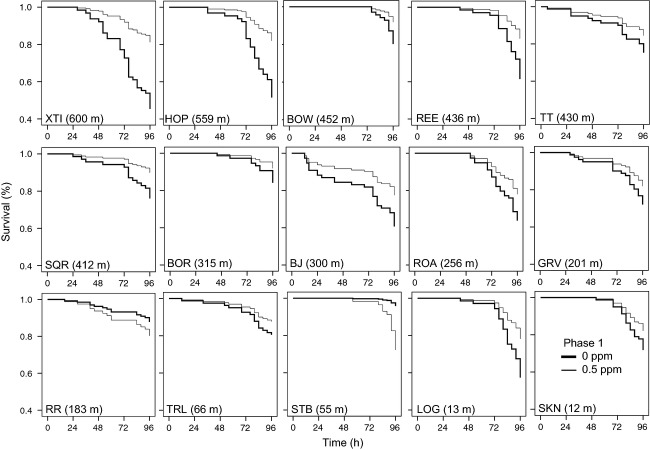
The proportion survival of wood frog tadpoles from 15 populations over time after being exposed to 0 vs. 0.5 mg/L of carbaryl at the hatchling stage and a lethal concentration of carbaryl (20 mg/L) as tadpoles. Values in parenthesis indicate the population's distance from agriculture.

We found significant population-level variation in the magnitude of induced tolerance to carbaryl (Fig.3; Table1). Cox regression analyses indicated four wood frog populations (HOP, LOG, REE, and XTI ponds), which are all relatively far from agriculture, displayed significant, induced tolerance to carbaryl. In these populations, exposed tadpoles had significantly higher tolerance to a lethal dose of carbaryl later in life than naïve tadpoles from the same population (all *P* < 0.05). In contrast, early exposure to 0.5 mg/L of carbaryl resulted in a decrease in tolerance for tadpoles from RR and STB pond, which are relatively close to agriculture, although only significant for STB (Fig.3; Table1).

**Table 1:** Hazard regression coefficient (b) of tadpoles exposed to 0 mg/L vs. 0.5 mg/L in Phase 1 determined by Cox regression analysis for tadpoles from each population. Censored values indicate % tadpoles that did not experience mortality by 96 h in the TTD assay. Bold values indicate a significant difference in the TTD of tadpoles exposed to 0 mg/L vs 0.5 mg/L

Population	% Censored	Hazard regression coefficient (*P*-value)
BJ	69	−0.7 (0.07)
BOR	88	−0.72 (0.23)
BOW	86	−0.95 (0.09)
GRV	77	−0.49 (0.24)
HOP	67	−1.20 **(0.001)**
LOG	68	−0.81 **(0.03)**
REE	72	−0.97 **(0.02)**
ROA	71	−0.59 (0.12)
RR	84	0.53 (0.30)
SKN	77	−0.52 (0.22)
SQR	83	−0.94 (0.06)
STB	84	2.04 **(0.001)**
TRL	84	−0.49 (0.33)
TT	80	−0.52 (0.25)
XTI	63	−1.3 **(<0.001)**

The magnitude of induced pesticide tolerance in a population was related to distance to agriculture. Using all 15 populations, we found a positive relationship between distance to agriculture and inducible tolerance (*r *= 0.56, *P* = 0.01; Fig.2B). The relationship between distance to agriculture and induced tolerance was stronger when the two populations with positive hazard regression coefficient (b) were excluded (*r *= 0.73, *P* = 0.002). Collectively, these results demonstrate that populations farther from agriculture were more inducible for carbaryl tolerance than populations closer to agriculture.

### Relationship between the magnitude of induced tolerance and naïve tolerance

Our final analysis examined the relationship between naïve pesticide tolerance and the magnitude of induced pesticide tolerance. Regardless of whether populations with positive b-values were included or not (Fig.2C), we found a negative relationship between the naïve tolerance of populations and the magnitude of induced tolerance in these populations (*r *= −0.73, *P* = 0.001 and *r *= −0.63, *P* = 0.01, respectively).

### Reaction norms of tadpoles exposed to sublethal vs. no carbaryl across 15 populations

**Figure 4:**
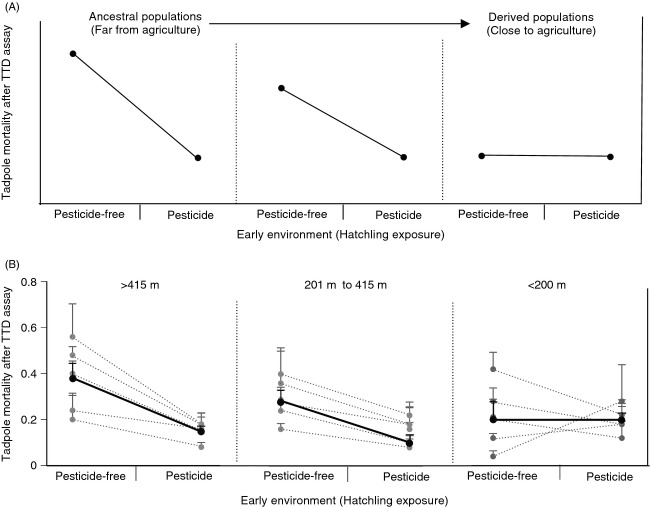
(A) Predicated reaction norms for ancestral and derived population if genetic assimilation (GA) is occurring. (B) Reaction norm of 15 wood frog populations that vary in distance to agriculture is consistent with predictions of GA. Dotted lines indicate the reaction norm for each individual population within a distance to agriculture range (i.e. >415 m, 201–415 m, or <200 m). Solid lines indicate the average reaction norm of all populations within a distance to agricultural range. Pesticide free = 0 ppm carbaryl and Pesticide = 0.5 ppm carbaryl.

Finally, using mean mortality of each population at the end of the TTD assay, we present our data in reaction norm format. We first indicate predicted reaction norms for ancestral and derived population if genetic assimilation (GA) is occurring (Fig.4A). Next, we present the reaction norm of each of the 15 wood frog populations (Fig.4B). Given the large number of populations, we report the reaction norms of all 15 populations by splitting the populations into three groups: Populations that are >415 m from agriculture (TT, BOW, REE, HOP, XTI), populations that are 201–415 m from agriculture (GRV, ROA, BJ, BOR, and SQR), and populations that are <200 m from agriculture (SKN, LOG, STB, TRL, and RR). In addition to reporting the reaction norm line for each population (dotted lines), we also report the average reaction norm of all populations within each group (solid lines). We find that the patterns of reaction norms of populations that vary in distance to agriculture are consistent with predictions of GA.

## Discussion

As human populations grow, there is a critical need to understand how anthropogenic stressors will influence the ecology and evolution of wild populations (Goudie 2005; Lawrence et al. 2012). In particular, there has been an increasing emphasis on identifying the underlying mechanisms that drive evolutionary responses of populations to anthropogenic influences, including the role of plasticity (Crispo et al. 2010). We found that population-level patterns of pesticide tolerance in wood frogs were consistent with evolutionary responses to agricultural pesticide use and that the patterns of induced versus constitutive tolerance were consistent with predictions of genetic assimilation. Our results suggest that the ability to induce tolerance to pesticides is a common phenomenon across multiple wood frog populations allowing amphibians to rapidly induce increased pesticide tolerance within a single generation. Additionally, patterns of inducible tolerance suggest that genetic assimilation may play a role in the evolution of constitutive tolerance in wild populations.

As our reliance on pesticides intensifies, there is a great need to understand the mechanisms that drive evolutionary responses of wild populations that are often inadvertently exposed to contaminant stressors. Toward this goal, one challenge in tracking evolutionary responses in wild populations is that the process can occur across broad temporal scales. However, using a space for time approach, it is possible to infer evolutionary processes. For instance, evolutionary theory predicts that if the ancestral state of the populations is to have inducible tolerance, then populations consistently exposed to pesticides (i.e. populations close to agriculture) should evolve high, constitutive tolerance whereas those not consistently exposed to pesticides (i.e. populations far from agriculture) should retain a low level of naïve tolerance that can be increased via induction by a sublethal exposure to the pesticide (Crispo 2007; Brausch and Smith 2009; Cothran et al. 2013). In support of this theory, we provide evidence demonstrating that wood frog populations living closer to agriculture had higher naïve tolerance to the insecticide carbaryl compared to those living far from agriculture. A growing number of studies have also demonstrated similar patterns in target and nontarget species (Brausch and Smith 2009; Cothran et al. 2013; Bendis and Relyea 2014; Nkya et al. 2014). For instance, a negative relationship between distance to agriculture and naïve tolerance was detected in fairy shrimp in response to cyfluthrin, methyl parathion, and DDT (Brausch and Smith 2009). A similar relationship was documented in mosquito populations in response to the pyrethroid insecticide, and deltamethrin (Nkya et al. 2014). Thus, despite the initially detrimental effects of pesticides on nontarget populations, evidence suggests that populations can evolve in response to these stressors. Identifying the mechanisms that drive these processes is an essential next step in understanding the broader implications of evolved tolerance in nontarget populations.

Evolved pesticide tolerance can be achieved through selection for existing constitutive traits or by the evolution of constitutive traits from initially plastic traits (i.e. genetic assimilation). If genetic assimilation plays a role in evolved constitutive tolerance, theory predicts that 1) ancestral populations (i.e. populations far from agriculture) should induce increase tolerance when exposed to pesticides, thereby making their formerly cryptic variation for pesticide tolerance become apparent to selection (i.e. noncryptic), 2) derived populations (i.e. populations close to agriculture) should express high naïve tolerance to pesticides, and 3) the degree of naïve tolerance will be negatively related with the magnitude of induced tolerance. Our analyses indicated that early exposure to sublethal carbaryl indeed induced increased tolerance to lethal concentrations of carbaryl in tadpoles from populations relatively far from agriculture (HOP, LOG, REE, and XTI ponds). Tadpoles with induced tolerance to carbaryl have previously been shown to have increased AChE concentration (Hua et al. 2013b). However, the molecular mechanisms allowing organism to achieve tolerance can be highly variable (i.e. modification of ACh binding sites, metabolic detoxification; Feyereisen 1995), underscoring the need for future studies exploring the mechanisms underlying induced tolerance. We also found that the magnitude of induced tolerance was positively correlated with proximity to agriculture. Consistent with our predictions of genetic assimilation, we found a negative relationship between induced pesticide tolerance and naïve tolerance; populations with high naïve tolerance to carbaryl expressed low amounts on induced tolerance to carbaryl. The results are consistent with our past study, which examined only four populations and found that the two populations closer to agriculture were unable to induce tolerance while those farther from agriculture were able to induce increase tolerance (Hua et al. 2013b). Thus, our results suggest that the ability to induce tolerance to pesticides may be a common phenomenon across multiple wood frog populations and patterns of inducible tolerance were consistent with predictions of genetic assimilation.

Within the past few decades, the field of evolutionary biology has seen a surge of interest in understanding the role of plasticity and genetic assimilation in evolutionary innovation (Wund 2012; Schlichting and Wund 2014). Understanding the ability for populations to induce pesticide tolerance and develop constitutive tolerance through the process of genetic assimilation may have broad conservation implications. Amphibian populations are declining worldwide, and pesticides are often implicated as a contributor to these declines (Blaustein et al. 2011). We suggest that genetic assimilation offers an alternative perspective to understanding the role of pesticide contaminants in amphibian populations declines. With growing human populations, genetic assimilation may allow wild populations to rapidly evolve in response to anthropogenic stressors in their environment. However, although genetic assimilation offers a potentially optimistic outlook for populations faced with anthropogenic stressors, consideration of potential costs associated with genetic assimilation will be crucial.

Although the evolution of constitutive trait expression can be achieved through selection for existing constitutive traits and selection on plastic traits (i.e. genetic assimilation), the role of genetic assimilation has largely been ignored. To date, our study provides one of the most comprehensive approaches examining the role of plasticity and genetic assimilation in the evolution of pesticide tolerance in wild populations. However, we emphasize that additional studies are imperative. The present study focused on a single species and pesticide; future work needs to expand to other taxa and pesticides to determine whether the identified patterns are generalizable. Further, our work does not consider the potential for nongenetic inheritance due to epigenetic inheritance (transmission of DNA methylation variants), somatic inheritance (parentally derived somatic resources that affect development), or behavioral inheritance (parental influence on developmental environment) and future work considering these contributions are necessary. Additionally, despite the many benefits of space for time substitutions for species with relatively longer generation times, future studies utilizing alternative model organisms with shorter generation times in controlled laboratory or mesocosms settings will be helpful in providing direct mechanistic evidence for genetic assimilation (Fox and Wolf 2006). Thus, while our study provides evidence consistent with the predictions of genetic assimilation in nature, a critical next step is to experimentally track and document this process across multiple generations. In addition to multigeneration research, future studies should also consider the standing intra-population-level variation, which can play a large role in determining patterns of local adaptation. Further, predictions of genetic assimilation infer that costs of plasticity drive selection for constitutive tolerance across time (Crispo 2007). Thus, an important next step is to identify these potential costs. Finally, future studies that identify the underlying molecular mechanisms associated with tolerance can potentially lead to the development of effective detection tools for predicting the contribution of genetic assimilation to the evolution of wild populations. In conclusion, plasticity and genetic assimilation offer an exciting and novel perspective for exploring how anthropogenic stressors influence the evolution of populations in nature (Schlichting and Smith 2002; Schlichting and Wund 2014). Studies exploring these mechanisms are imperative and will likely have broad evolutionary and conservation implications.

## Data archiving statement

Raw data of the main results are available from the Dryad Digital Repository: http://dx.doi.org/10.5061/dryad.82bg7.

## Supporting Information

**Appendix S1.** Additional supplementary results. **Table S1.** Anuran egg collection data and initial masses of the tadpoles used in the experiment. **Table S2.** Results of the regression analysis for the relationship between distance from agriculture and naïve tolerance to carbaryl, distance to agriculture and plasticity to carbaryl, and plasticity to carbaryl and naïve tolerance to carbaryl. **Figure S1.** Mass of wood frog tadpoles from 15 populations after being exposed to 0 vs. 0.5 mg/L of carbaryl at the hatchling stage in *Phase 1* of the experiment. **Figure S2.** Proportion mortality (mean ± SE) of wood frog tadpoles from 15 populations (organized from highest tolerance to lowest) after being exposed to 0 mg/L of carbaryl at the hatchling stage and after 96 h of exposure to 20 mg/L of carbaryl as a tadpole.
