# Supplementary material for: The contribution of phenotypic plasticity to the evolution of insecticide tolerance in amphibian populations
Source: Evol Appl. 2015 May 27;8(6):586–96. doi: 10.1111/eva.12267 (PMC4479514; doi:10.1111/eva.12267)
Supplement: Supplementary file 1 [file eva0008-0586-sd1.docx]

Appendix 1- Additional supplementary results

*Effect of sublethal exposure on tadpole mass*

To determine whether exposure to sublethal concentrations of carbaryl affected tadpole mass after the initial sublethal exposures (i.e. after *Phase 1*), we conducted an analysis of variance (ANOVA). Since the assumption of homogeneity was not met, we conducted the analyses on rank-transformed mass. We then conducted pairwise comparisons using SNK test, which is well suited for rank-transformed data (Quinn and Keough 2002).

Early exposure to sublethal concentrations of carbaryl during the hatchling stage had a significant effect on tadpole mass by the end of *Phase 1* (F_29, 270_ = 6.4 p < 0.001). While tadpoles from the populations varied in mass (Phase 1, 0 mg/L: 0.018 ± 0.001 g to 0.025 ± 0.001 g; Phase 1; 0.5 mg/L: 0.017 g ± 0.001 to 0.029 ± 0.001 g), SNK pairwise analysis indicated only one population (XTI; Fig 1) where the mass of tadpoles differed between those exposed to an early exposure to sublethal concentrations of carbaryl and those not exposed to carbaryl during Phase 1. In this population, tadpoles exposed to 0.5 mg/L as hatchlings were significantly heavier than tadpoles exposed to no carbaryl as hatchlings.

| **Pond** | **Pond ID** | **Date collected** | **# of egg masses** | **Latitude (N)** | **Longitude (W)** |
| --- | --- | --- | --- | --- | --- |
| Blackjack | BJ | 8 April | 10 | 41° 39.9' | 80° 30.8' |
| Boro | BOR | 11 April | 10 | 41° 55.2' | 80° 1.9' |
| Bowl | BOW | 11 April | 10 | 41° 55.6' | 79° 48.2' |
| Graveyard | GRV | 7 April | 10 | 41° 41.0' | 80° 2.8' |
| Hopscotch | HOP | 7 April | 10 | 41° 52.1' | 80° 28.0' |
| Log | LOG | 11 April | 10 | 41° 58.1' | 79° 36.1' |
| Railroad | RR | 6 April | 10 | 41° 36.4' | 80° 22.9' |
| Reed | REE | 11 April | 10 | 41° 58.9' | 79° 58.2' |
| Road | ROA | 11 April | 10 | 41° 53.1' | 79° 36.3' |
| Skinny | SKN | 11 April | 10 | 41° 59.9' | 79° 46.5' |
| Square | SQR | 6-8 April | 10 | 41° 50.5' | 80° 14.4' |
| Staub | STB | 7 April | 10 | 41° 35.4' | 80° 25.9' |
| Trailer Park | TRL | 4-5 April | 10 | 41° 34.1' | 80° 27.1' |
| Turkey Track | TT | 8 April | 10 | 41° 37.8' | 79° 54.7' |
| Xtine | XTI | 13 April | 10 | 41° 37.6' | 80° 27.7' |

Table A1. Anuran egg collection data and initial masses of the tadpoles used in the experiment.

| Regression | | r | r^2^ | p-value  (One-tailed) | p-value  (Two-tailed) |
| --- | --- | --- | --- | --- | --- |
| Distance to agriculture vs. Naïve tolerance | | -0.47 | 0.22 | 0.038 | 0.076 |
| Distance to agriculture vs. Plasticity to carbaryl | With STB and RR | 0.58 | 0.33 | 0.012 | 0.025 |
|  | No STB and RR | 0.74 | 0.54 | 0.002 | 0.004 |
| Naïve tolerance vs. Plasticity to carbaryl | With STB and RR | -0.74 | 0.55 | 0.001 | 0.002 |
|  | No STB and RR | -0.67 | 0.45 | 0.006 | 0.012 |

Table A2. Results of the regression analysis for the relationship between distance from agriculture and naïve tolerance to carbaryl, distance to agriculture and plasticity to carbaryl, and plasticity to carbaryl and naïve tolerance to carbaryl.

**Figure captions**

Figure A1. Mass of wood frog tadpoles from 15 populations after being exposed to 0 vs. 0.5 mg/L of carbaryl at the hatchling stage in *Phase 1* of the experiment.

Figure A2. Proportion mortality (mean ± SE) of wood frog tadpoles from 15 populations (organized from highest tolerance to lowest) after being exposed to 0 mg/L of carbaryl at the hatchling stage and after 96 hrs of exposure to 20 mg/L of carbaryl as a tadpole. Tadpoles from STB were significantly more tolerant than BJ, REE, LOG, HOP and XTI, and RR is significantly more tolerant than HOP and XTI.

Figures

Fig. A1

Fig. A2
